# Study on the incorporation of phase change material and differently embedded water circulation pipes for enhanced thermal management of photovoltaic system

**DOI:** 10.1038/s41598-025-18859-1

**Published:** 2025-10-13

**Authors:** Mohammad Arsalan Khan, Mohammad Uzair, Mohammad Mursaleen, Frank Wuttke, Manzoore Elahi M. Soudagar, Adham E. Ragab

**Affiliations:** 1 Geomechanics and Geotechnics Group, Kiel University, 24118 Kiel, Germany; 2 Department of Civil Engineering, Zakir Husain College of Engineering and Technology, Aligarh Muslim University, Aligarh, 202001 India; 3 Centre for Integrated Green and Renewable Energy, Zakir Husain College of Engineering and Technology, Aligarh Muslim University, Aligarh, 202001 India; 4 China Medical University Hospital, China Medical University (Taiwan), Taichung, 40402 Taiwan; 5 Centre for Promotion of Research, Graphic Era (Deemed to be University), Clement Town, Dehradun, India; 6 Centre for Research Impact and Outcome, Chitkara University Institute of Engineering and Technology, Chitkara University, Rajpura, Punjab 140401 India; 7 Department of Industrial Engineering, College of Engineering, King Saud University, 12372 Riyadh, Saudi Arabia

**Keywords:** Photovoltaic, Efficiency improvement, Phase change material, Thermal management, Latent heat improvement, Civil engineering, Mechanical engineering

## Abstract

This research presents an experimental investigation on the thermal management and improvement of electrical efficiency of photovoltaic (PV) systems employing a phase change material (PCM) and water combination technique as heat dissipation systems through an improved design. This experiment was conducted in the semi-arid environment of Aligarh, India. The experiment utilised an aluminium enclosure that housed PCM with a melting point of 30 °C (OM 30), along with integrated water circulation pipes. The study involved the evaluation and comparison of three types of PV systems: a PV system with an empty casing attached to the rear surface, a PV system with a casing filled with PCM, and a PV system with a casing filled with a combination of PCM and water circulated using embedded serpentine pipes (without making contact with the casing walls). The water flow rates used in the study were 0.0027 kg/s and 0.0034 kg/s. Power generation, exergy analysis, power efficiency, and performance metrics, specifically the power increase performance variable of the system were compared. The average increases in electrical efficiency, power output, power enhancement, and maximum average temperature reduction, were found to be 13.75%, 24.14 W, 27.6%, and 5.6 °C, respectively at water flowrate of 0.0034 kg/s (best case). The findings of the study indicate that the improvement in the design concept of the PCM and water combination system results in superior performance of the PV panel compared to other approaches.

## Introduction

Compared to the scenario of the past, the United States Energy Information Administration’s annual energy forecast for 2019 is that, the agency anticipated a 50% increase in global energy demand by 2050^1^. To meet this demand, approximately 28% of renewable energy and 72% of non-renewable energy resources will be utilised. Typically, environmental issues such as climate change and air pollution are exacerbated by technology that consumes a great deal of energy and is fuelled by non-renewable resources^2,3^. To lessen dependency on non-renewable energy sources, emphasis is being placed on generating energy from renewable sources^4^. At present, photovoltaic (PV) solar energy is the most widespread, unlimited, and pure form of renewable energy, which converts sunlight into usable electrical power and is one of the most extensively utilised techniques of producing renewable energy, reducing non-renewable sources’ usage^5^. However, solar photovoltaic (PV) technology is increasingly adopted for the production of electricity globally, owing to its scalability, decreasing costs, and technological advancements. The deployment of PV systems for electricity generation has seen significant growth. For instance, countries like Germany, China, and the United States have invested heavily in large-scale PV installations, contributing substantially to their renewable energy portfolios. This trend is driven by the need to diversify energy sources, reduce carbon emissions, and achieve energy independence. Moreover, the versatility of PV technology, which can be integrated into various environments such as rooftops, solar farms, and even portable devices, further supports its widespread use for electricity production^6,7^. With increasing operating temperatures in variable climatic conditions^8^, there are some drawbacks, such as low power output and low conversion efficiency^9^. As a result, photovoltaic systems are limited in their potential^10^. For every 1 °C increase in ambient temperature, the efficiency of photovoltaic decreases by 0.4% to 0.5%^11^. Hence, it is important to find innovative techniques for cooling solar PV panels that improve electrical efficiency, reduce the temperature of the panels’ surface, and prolong the longevity of solar-photovoltaic panels^12^.

Typically, heat from a photovoltaic panel is efficiently dissipated by utilising passive and active cooling techniques, which also aids in enhancing the optimum efficiency of the solar PV system^13^. Active cooling techniques require external power for heat dissipation, which also adds to the cost of use and complicates the system by adding additional fans, pumps, ducts, and heat sinks^14^. Although passive cooling techniques are effective, they require no external power or energy for heat dissipation. This occurs naturally. Consequently, emphasis is put on passive cooling techniques.

## Literature review

Numerous research studies have been conducted to reduce the surface temperature of photovoltaic panels through passive cooling techniques A study by Sheikholeslamiet al.^15^ has investigated the environmental and energy performance of PVT systems combined with reflectors, nanofluid filters, and thermoelectric generator (TEG) layers, highlighting the potential for increased solar energy absorption and enhanced thermal management. The inclusion of nanofluids, which serve as spectral splitters, allows for better utilization of the solar spectrum by filtering specific wavelengths, thus improving the efficiency of both thermal and electrical energy generation. Although much effort has been expended on using phase-changing materials. Due to the uniqueness of their thermophysical properties, it is a novel material used in passive cooling strategies. It has been extensively accepted to regulate the high operational temperature of photovoltaic system in diverse and stable climates^16^. During the phase transition, phase transition materials can either retain or release heat energy. When phase change material (PCM) reaches its melting point, it absorbs perceptible heat first, followed by latent heat, causing it to continue melting and vice versa. The integration of PCM and PV panels, known as PV-PCM systems, was first introduced in 1978 to minimise the surface temperatures of PV panels^17^. Recent research findings that are highly relevant to the current investigation of the PV combined with PCM system in various climates and the behaviour of PCM material have been highlighted. A study by Sheikholeslami^18^ has showcased the potential of Nano-Enhanced Phase Change Materials (NEPCM) in solar energy systems, particularly through the innovative application of a honeycomb configuration for thermal storage enhancement. This research explores the utilization of various solid materials, including Stainless steel (SS), Aluminum-6061-T4 (Al-6061), and pure aluminum (Al), in conjunction with NEPCM filled honeycomb structures. The findings reveal that thinner honeycomb edges and the use of pure aluminum significantly expedite the melting process, suggesting promising implications for solar energy applications. This study underscores the relevance of NEPCM and its tailored configurations in advancing the efficiency and performance of solar energy systems. Ho et al.^19^ conducted a simulation study on building-integrated photovoltaic systems (BIPV) by integrating the MEPCM (Microencapsulated Phase change material) layer and a conventional photovoltaic system as a standard and comparing their performance. According to their findings, the average electrical efficacy of a system with a MEPCM layer was 19.61%, which was 2.1% greater than that of a system without MEPCM. Stropnik and Stritih^20^ made another attempt by conducting an experimental and numerical simulation of roof-top PV combined with a PCM system using the organic phase change material (PCM-RT28HC) in Slovenia during the month of October. Using PCM led to a 2.8% increase in electrical conversion efficiency and a 9.2% increase in power output, according to experimental results. The author has deduced that the implementation of a PCM system in photovoltaic (PV) panels resulted in a rise of 0.5% to 1% in power output and an increase of 4.3% to 8.7% in electrical efficiency, following a reduction of 14.4 °C in the surface temperature of the PV panels. Hasan et al.^21^ continued a similar endeavour and experimented with the warm climate of the United Arab Emirates. The annual performance of photovoltaic (PV) panels was evaluated using the organic paraffin wax phase change material from the RT line, specifically RT42. The authors asserted that a 13 °C decrease in maximum PV panel temperature during April and incorporating organic PCM RT42 on the back of the photovoltaic test rig led to a 5.9% increase in power generation year-over-year. Due to incomplete freezing at night and partial melting during the day, the PCM was also found to be ineffective during the warmest and coldest months. Choubineh et al.^22^ recently conducted an investigational study on the effectiveness of inorganic salt hydrates PCM 32/280 in enhancing the performance of air-cooled PV panels in Iran. For thermal regulation of the PV, forced air circulation at three mass flow rates: 1.05 kg/s, 0.95 kg/s, and 0.75 kg/s, and natural air circulation with and without a combination of inorganic salt hydrates were used. The PV modules’ average and maximum reduction temperatures were determined to be 4.3 °C and 10.0 °C, respectively, for natural air circulation. They forced air with flow rates of various low velocities in convection mode. When using the PCM under natural and forced air convection, the system’s overall performance efficiency increases to approximately 9%. An experimental study was conducted by Sandro et al.^23^ in Croatia, under Mediterranean climate conditions, to compare the performance of two different 20 Wp PV PCM systems. One system had a full-size PCM-filled container attached at the back of the panel, while the other had a smaller PCM-filled container attached at the back of the panel. The overall efficiency of the systems was compared, and it was found that the overall efficiency of the system with the small PCM container was about 10.7% compared to the reference. However, the full-size PCM container’s performance was only 2.5%. However, the study did not clearly mention the gaps between the PCM containers and their effect on uniform heat dissipation. This issue may affect the overall heat transfer process and the potential impact on the overall efficiency of the system. A study conducted by Al Miaari et al.^24^ focused on enhancing the cooling efficiency of rooftop PV panels in the hot climate of Dharan, Saudi Arabia. They selected a 20 Wp monocrystalline PV panel and employed RT 42 phase change material (PCM) with a temperature range of 42 °C. The researchers strategically placed six small, detachable aluminium containers filled with PCM behind the PV panel. The results indicated a notable 5.23% improvement in the system’s power performance compared to the reference. Arici et al.^25^ Conducted an in-depth study on the integration of phase change materials (PCM) with photovoltaic (PV) panels, presenting a simplified numerical model to optimize the PCM layer and assess the economic viability of such systems. The study found that PCM can significantly lower PV panel operating temperatures by up to 10.26 °C, leading to an efficiency improvement of up to 3.73%. This reduction in temperature and subsequent efficiency gain underscores the technical viability of PCM for cooling PV panels. However, the study also identified critical economic challenges, noting that current PCM costs are too high to justify their widespread use without significant price reductions. Additionally, improved thermal management of the PCM layer is essential to maximize efficiency gains and make the system economically feasible. The findings suggest that while PCM technology holds promise for enhancing PV panel performance, further advancements in PCM cost and thermal design are necessary to achieve practical and economical implementation. Another work conducted by Arici et al.^26^ In their study, they investigated the impact of boundary conditions (BCs) on the natural convection of molten phase change material (PCM) inside enclosures and examined the effects of different BCs on the flow and heat transfer characteristics of PCM for cooling photovoltaic (PV) systems. The numerical study focused on a rectangular enclosure with liquid PCM at a 30° inclination and explored various Biot numbers, aspect ratios, and Rayleigh numbers. It was found that the Biot number significantly affects the mean Nusselt number and convective heat transfer: low Biot numbers restrict convection currents, while high Biot numbers enhance heat transfer. These insights are crucial for optimizing thermal management in PV systems using PCM, ensuring effective heat dissipation and improved system performance. Talele et al.^27^ investigates the use of phase change materials (PCMs) to delay thermal runaway trigger points in lithium (Li)-ion batteries. Results show that PCM submersion significantly delays trigger points, enhancing safety. However, fire propagation remains a concern due to PCM volatility. Insulation at PCM walls is recommended to mitigate propagation, highlighting the importance of PCM-equipped passive cooling strategies. A study conducted by Sheikholeslami et al.^28^ investigated the integration of phase change materials (PCMs) and nanofluids within a concentrated photovoltaic thermal (PVT) system to enhance its thermal and electrical performance. They explored the impact of different fin configurations and nanoparticle dispersions on heat absorption and temperature regulation. Their numerical simulations revealed that the short base arrow fin configuration, combined with multi-walled carbon nanotube (MWCNT) nanoparticles, optimized heat dissipation and improved electrical efficiency by up to 3.72%. This study underscores the potential of advanced cooling techniques using PCMsand^29^ conducted a detailed numerical investigation to examine the impact of various heated wall configurations on the melting process of phase change materials (PCMs) within a rectangular cavity filled with RT-27. The configurations studied included square, rectangular, trapezoidal, and curvy shapes. Their analysis revealed that the geometry of the heated walls significantly influences the melting process, with curvy wall configurations showing the most improvement. Specifically, curvy walls enhanced free convection, reduced flow separation, and promoted uniform fluid motion, resulting in a 57.6% reduction in melting time and a 16.3% increase in energy storage capacity compared to the baseline case. Sheikholeslami et al.^30^ investigated the improvement of PV system performance using a phase change material (PCM) and water combination for cooling. They incorporated fins and graphene nanoparticles within paraffin (RT25) to enhance heat absorption and uniformity of cell temperature. The results demonstrated a 29.53% increase in temperature uniformity and a significant enhancement in electrical performance, with a 10.22% improvement for coated glass, highlighting the efficacy of PCM in managing thermal loads and boosting PV efficiency.

However, the study still overlooked addressing a significant issue: the still noticeable gap between the containers and the uniform heat dissipation, which could potentially impact the overall heat transfer process. The major problems that arise with PCMs, especially inorganic PCM materials, are supercooling, low thermal conductivity, and corrosive nature. But organic PCM materials typically have issues with supercooling and low thermal conductivity^31^, which prevent them from reaching their full potential. To address these shortcomings, researchers have employed nanomaterials, high thermal conductivity heatsinks and the phase change material added with a coolant water pipe^32^. The most promising is water and PCM combination which does not need a complicated preparation of fluid such as with the nanofluid material.

In addition to research on PV-integrated PCM (PV-PCM) systems, there are relevant studies on photovoltaic cooling using water circulation heatsinks and a combination of water and PCM heatsinks. Bahaidarah et al.^33^ conducted a computational and experimental analysis to determine the efficiency of the PV module in Dhahran’s humid climate by circulating water at the back. The authors concluded that using water circulation cooling technology decreased the maximal operating temperature of the module from 45 to 34 °C, decreased the module’s overall temperature by nearly 20%, and increased the module’s electrical efficiency by about 9%. Similarly, the authors^34^ present a comprehensive experimental and computational analysis for cooling a PV panel utilising a cooling procedure based on using a convergent heat exchanger channel in Dharan, Saudi Arabia. As opposed to the uncooled photovoltaic, the operational temperature of the cooled photovoltaic panel decreased from 71.2 °C in summer to 45.1 °C and from 48.3 to 36.4 °C in winter. For summer action, PV panels’ power output and electrical efficiency increased by 35.5%. In contrast, for winter operations, they increased by 26.1% and 36.6%, respectively, and their computational and experimental observations are in agreement.

**Fig. 1:**
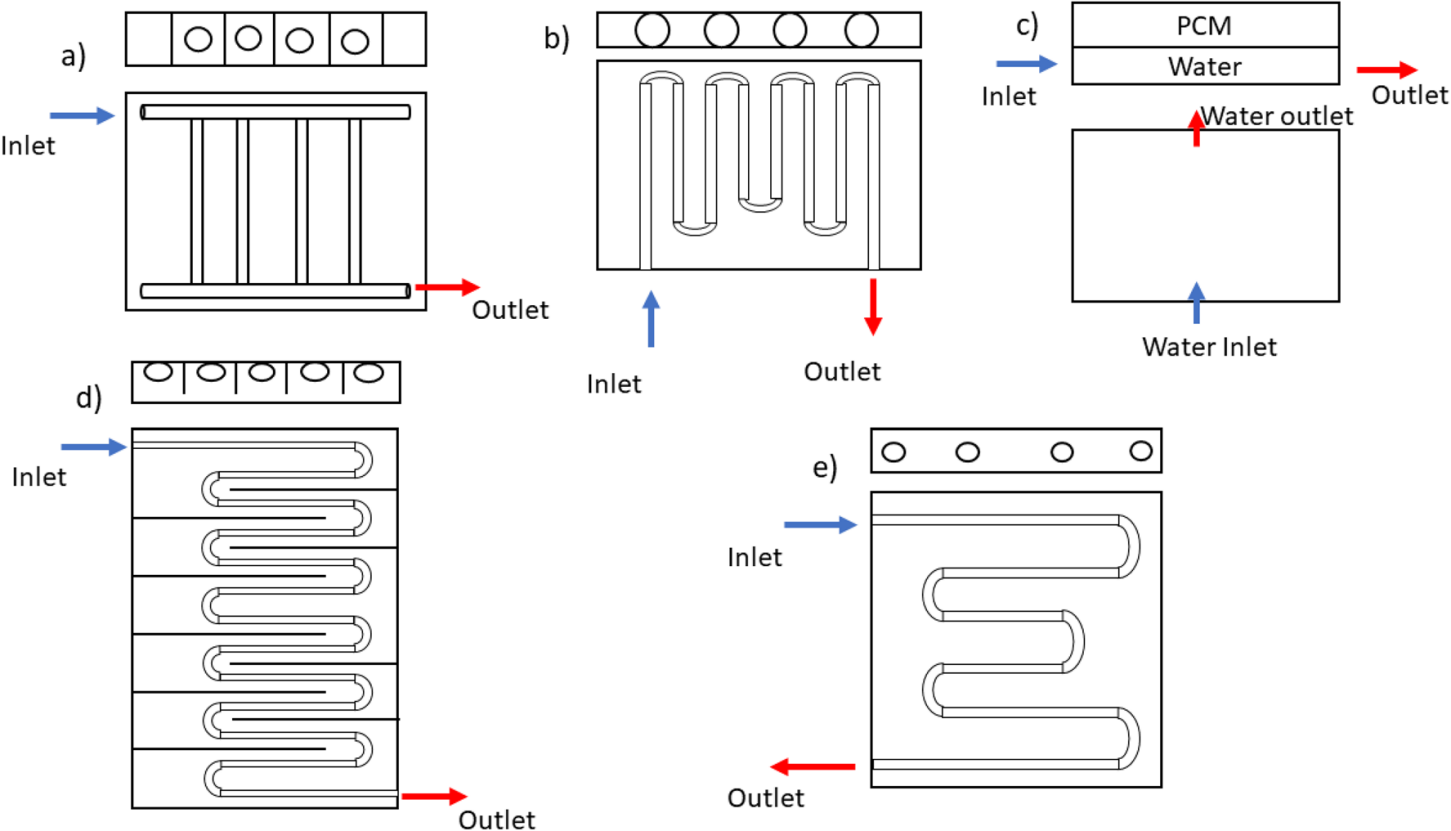
Schematic views and the comparison of the previous literature’s different PV cooling system design with Present study, typical sketches drawn to represent study design of: (**a**) Preet et al.^35^, (**b**) Kazemien et al.^41^, (**c**) Sudhakar et al.^42^, (**d**) Xu et al.^36^, (**e**) Present study.

In a semiarid climate, extraordinary research progresses towards the novel combination of water and PCMs heatsink for cooling photovoltaic panels as key researches are sketched up in Fig. 1; this strategy was simple and easy to handle as compared to other fluids. In the proposed design as shown in Fig. 1e, the cooling pipe is entirely contained within the PCM and not directly attached to the casing. This allows for even heating, better melting and solidification of the PCM, and reduces thermal resistivity. Therefore, the thermal performance is improved over the traditional designs. Preet et al.^35^ experimented with the Indian composite climate to determine the effectiveness of cooling photovoltaics with water and the combination of water and PCM heat sinks, where PCM is filled in special aluminium finned casings with copper pipes and pipes touching the casing internally, and three water flow rates; 0.013 kg/s, 0.023 kg/s, and 0.031 kg/s; and paraffin wax (RT30) materials. Using a water discharge rate of 0.031 kg/s led to a maximum temperature reduction of 47%, while PCM and water led to a maximum temperature reduction of 53%. Consequently, the electrical efficacy of both systems is increased by a maximum of 10.66% and 12.6% compared to the conventional PV system. However, in a recent study by Xu et al.^36^ the metal fins in the PCM container, rapidly melt PCM due to increase of the area of fins and PCM is limited in their potential in their initial condition. Kazemien et al.^37^ examined glazed and unglazed photovoltaic thermal systems (PVTs) equipped with a phase change material (PCM) layer, utilizing various coolant fluids pure water, a water/ethylene glycol (EG) blend at 50%, and EG at 100%. Notably, their system design featured an internally integrated fluid flow pipe within the casing, making direct contact with its surface. Their findings revealed that employing a water/EG mixture decreased thermal energy efficiency but significantly enhanced thermal exergy efficiency. However, the introduction of EG at 50% to pure water and EG at 100% led to reduced energy loss reductions, recording values of 23.33% and 48.58%, respectively, in contrast to the 9.28% reduction observed in the pure water case, all compared to unglazed systems. Despite the contact between the internal pipe and casing, the study favoured the water/EG mixture as a superior coolant for colder climates compared to pure EG. A study conducted by Sheikholeslamiet et al.^38^ explored the impacts of environmental parameters and dust deposition on the efficiency of photovoltaic/thermal (PVT) systems with a trapezoidal cooling channel incorporating ternary nanofluid (Fe_3_O_4_–TiO_2_–GO in water). The study found that increasing ambient temperature boosts thermal performance by 33.46%, while dust deposition and higher wind speed reduce PVT efficiency by 16.6% and 13.14%, respectively. The presence of fins and a thermoelectric generator (TEG) layer enhanced electrical output. The research highlights that ternary nanofluids and optimized cooling configurations can significantly improve PVT system performance and CO_2_ mitigation. In a recent study conducted by Sheikholeslamiet et al.^39^ an innovative design for heat transfer tubes in photovoltaic/thermal (PVT) systems was investigated to enhance electrical and thermal efficiency. The research utilized graphene nanoplatelet (GNP) nanofluids to improve heat transfer. Results showed that an 8-lobed tube design, combined with GNP nanofluids at a 0.1% weight fraction, significantly improved the system’s performance. Specifically, electrical efficiency increased by 5.8%, thermal efficiency reached 55.22%, and exergy efficiency was 15.32% at a Reynolds number of 1611. This study highlights the effectiveness of nanofluids in optimizing PVT system performance. A study conducted Subhedar et al.^40^ a computational investigation focuses on enhancing the thermal performance of electric vehicle (EV) battery packs using liquid cooling. Al_2_O_3_/EG-water dispersion is utilized as the cooling medium in an immersion cooling system, with a range of volume fractions tested. Results show that a volume fraction of 4% and above of Al_2_O_3_/EG-Water Nano-coolant effectively maintains battery temperature below 50 °C, mitigating degradation and thermal runaway. These findings underscore the efficacy of Nano-coolants in indirect liquid cooling battery thermal management systems, particularly for high discharge rates, aligning with the objectives of PV-PCM-water combination thermal management.

Sudhakar et al.^42^ attempted yet another innovative approach. Here, a cooling system is established and embedded behind the PV panel surface using a combination of phase change materials (organic OM-35) and natural water flow (up to down flow regime and vice versa) to mitigate the high operating temperatures of the PV in Chennai’s climate. The up-to-down water flow regime strategy was more effective, resulting in a 5.4 °C decrease in average temperature and increased electrical efficiency and power output of 12.4% and 13.54%, respectively.

The complexities of effectively regulating high temperatures within PV panels have been highlighted in the literature, leading to the identification of various cooling methods. One promising approach involves combining PCM and water. However, a common issue in literature is the use of internal water flow pipes touching the PCM casing, which limits optimal heat transfer from PCM to water. Aside from the thermophysical properties of PCM, certain PCMs have issues such as supercooling or incomplete charging and discharging throughout the day and night, which either directly or indirectly impact the overall life of the PV cooling system. In addition to thermal improvements, the proposed non-contact pipe design offers several broad advantages. First, the built-in pipe ensures efficient heat extraction, reducing the total PCM volume required without compromising cooling performance, thereby reducing material costs. Second, this design avoids welding or bonding to the casing walls, thereby simplifying construction and making maintenance and the modular configuration of the in-built pipe of PCM casing unit provides easy scalability to larger PV arrays, which is important for industrial or utility-scale adoption.In light of this, the design of the system must be improved to effectively address this issue related to PCM heat exchange and overall performance of the system.

### Research gap and novelty

Therefore, a new sustainable integrate system is proposed that should facilitate heat management from the PCM casing (attached to the rear of PV panel). For this, a serpentine pipe is integrated into the casing that runs through the inside of the casing box, so as to facilitate heat exchange from the PCM also, this design of casing is easy to handle and assemble. Numerical study of Khan et al.^43^ indicated that if the boundary of the pipe touches the wall of casing, the heat transfer will be localised for the casing wall. Therefore, in this study, the pipe is constructed without touching the boundaries of the casing so to maximise the heat transfer from the PCM. That is, the transfer of heat from the PV panel takes place from rear of the PV panel to the PCM via a layer of attached casing; the heat from the PCM is then directly absorbed by the boundaries of the pipe and transferred to the fluid flowing through the pipe.

The objective of the current study is to experimentally analyse the electrical performance of the rooftop PV system under semiarid environmental conditions. Experimentation is conducted in several phases. The first stage is to connect the PV panel to an aluminium casing of the same size as the PV panel, which is integrated with a serpentine-shaped aluminium absorber pipe without directly touching the container walls (internal) as shown Fig. 1e. In the second step, the aluminium casing is filled with PCM material to maintain the temperature of the PV. In the final step, water is pumped through serpentine absorber pipes at varying flow rates, extracting heat from the PCM and increasing its heat-storage capacity. This pipes or conduit’s primary purpose is to enhance the capacity of the given PCM to store heat by conducting the extra heat away. A reference PV panel is kept parallel to the cooling PV system for comparison purposes. Several parameters, including power generation, analysis of exergy, power efficiency, for the photovoltaic, and performance metrics like power increase performance variable were calculated and compared based on the findings of the experiments. The Novelty of this work lies in the design, integration and experimental validation of the proposed system.

1. Innovative serpentine Pipe integration: The conventional PV-PCM cooling system have pipes/tubes that are externally or indirectly attached the casing walls, this study implements an insert serpentine pipe inside of the PCM filled casing without direct wall contact, which optimize heat transfer to the PCM for uniform uptake, and was free from heat transfer localized heating as indicated by Khan et al.^43^
2. PCM/Water Hybrid Cooling System: The serpentine conduit is not just an integration that allows for simultaneous heat transfer while also storing in the PCM while providing the opportunity to transfer excess heat away to circulating water. The circulating water also improves the effective storage capacity of the PCM.
3. Experimental Validation under Semi-arid climate Condition: The most of the previous PV PCM study was numerical or has been tested in temperate climates. This work includes an experimental assessment of the integrated system in semi-arid rooftop conditions which presents novel findings on PCM efficacy in high irradiation and changing temperature situations.

In summary, the contribution of the study is not only to replicate PCM for PV cooling, but also to propose and test a novel, hybrid design that is more likely to be sustainable, does not require complex assembly or fabrication, extends the utility of PCM in the experimental phase, demonstrates improvements in PV efficiency, and finally establishes context-specific performance analysis in semi-arid climate regions.

## Theoretical analysis

The energy analysis provided by Kazemien et al.^37^ and Sudhakar et al.^42^ evaluated the energy balance of a conventional PV panel and the cooling of a panel made with an innovative PCM material with water combination.

Taking into account the different system (PV, PV/PCM, and PV/PCM with water circulation) as a control volume and under the assumption of a steady state, the energy equation for the PV/PCM with water circulation system can be formulated as follows:

1 $$\Sigma \dot{E}_{\text{in}} =\Sigma \dot{E}_{\text{out}}+\dot{E}_{\text{loss}}$$

2 $$\dot{E}_{\text{sun}}+\dot{E}_{\text{mass,in}}=\dot{E}_{\text{el}}+ {\dot{E}}_{el}+{\dot{E}}_{mass, out}+{\dot{E}}_{loss}$$

${\dot{E} }_{in}$,${\dot{E}}_{out}$, and ${\dot{E}}_{loss}$ denote the rates of energy associated with input, output, and losses, respectively, within the considered systems. Within this formulation, the term $\dot{E}$_sun_ corresponds to the received rate of useful solar irradiation, denoted as $\dot{G}$, impacting the PV/PCM-water circulation system, and this can be calculated as (3):

3 $$\dot{G}={\dot{G}}_{beam}+ {\dot{G}}_{diffuse}$$

${\dot{G}}_{beam}$ and ${\dot{G}}_{diffuse}$ represent the beam and diffuse segments of solar irradiance, respectively.

${\dot{E}}_{el}$ represents the electrical power output from the systems (PV, PV/PCM, and PV/PCM with water circulation), and its calculation follows the subsequent Eq. (4):

4 $${\dot{E}}_{el}={V}_{oc}\times {I}_{sc}\times FF$$

${V}_{oc}$ and ${I}_{sc}$ denote the open circuit voltage and short circuit current of the PV unit, respectively, while FF (fill factor) is also considered in the equation.

Equations (1, 2) is used to compute the energy associated with the mass flow rate as follows (5):

5 $${\dot{E}}_{th}={\dot{E}}_{mass,out}- {\dot{E}}_{in}={\left(\dot{m}{C}_{p}\right)}_{f}\cdot {({T}_{out}-{T}_{in})}_{f}$$

considering parameters such as $\dot{m}$, ${C}_{pf}$, ${T}_{in,f}$, and ${T}_{out,f}$. These variables denote the mass flow rate through the collector, the specific heat capacity of the working fluid, and the inlet and outlet temperatures of the working fluid, respectively. However, in the specific scenario where PCM heat is extracted by a water circulation pipe, the system’s useful thermal energy absorbed by the working fluid can be represented distinctly, emphasizing the role of the water circulation in extracting heat from the PCM.

The overall energy of the system can be acquired through the following method:

6 $${\dot{E}}_{ov}={\dot{E}}_{el}+ {\dot{E}}_{th}$$

### Data reduction

Usually, using a lot of data leads to inaccurate results. Data-reduction methods are employed to address this issue. For the PV panel with all cooling cases, electrical efficiency was employed by Eq. (7) and thermal efficiency of PV-PCM with water circulation was employed by Eqs. (8)^37^

7 $${\eta }_{elect}=\frac{{I}_{sc}{V}_{oc}FF}{{A}_{s}G}$$

8 $${\eta }_{th}=\frac{{\dot{E}}_{mass, OUT-}{\dot{E}}_{mass, IN}}{{\dot{E}}_{sun}}=\frac{{\dot{m}}_{f}.{c}_{pf}.{\left({T}_{out}-{T}_{in}\right)}_{f}}{{A}_{s}G}$$

where $\dot{G}$ represents sun irradiance (W/m^2^), A_s_ gives the surface area of solar panel (m^2^), 0.7 is the value of fill factor (FF), ${V}_{oc}$ and ${I}_{sc}$ represents open circuit (maximum) voltage (V) and short circuit current (A).

Moreover, the overall energy efficiency of the PV system with PCM and water was computed as (9):

9 $${\eta }_{ov}={\eta }_{th}+{\eta }_{el}$$

In evaluating the electrical power needed for fluid pumping the following equation provided by reference^41^.

10 $${\dot{E}}_{net,pump}= {\dot{E}}_{el}- {\dot{E}}_{pump}$$

In the context of this analysis, ${\dot{E}}_{net, pump}$ represents the net electrical power output of the PV/PCM system with water circulation, ${\dot{E}}_{el}$ denotes the calculated electrical power output of the system using Eq. (4), and ${\dot{E}}_{pump}$ signifies the electrical power required for pumping as determined by the following calculation:

11 $${\dot{E}}_{pump}=\frac{\dot{m}\Delta P}{{\rho }_{f}{\eta }_{pump}}$$

where $\Delta P$ represents the pressure drop in the PV/PCM water flow pipe, and ${\eta }_{pump}$ denotes the efficiency of the pump, the calculation of the net electrical efficiency of the PV/PCM with water circulation system is derived as follows^44^:

12 $${\eta }_{net}=\frac{{\dot{E}}_{el}- {\dot{E}}_{pump}}{{A}_{s}G}$$

### Thermodynamic analysis

From the perspective of exergy, the effectiveness of photovoltaic without PCM and photovoltaic with PCM/water (PV PCM/water panel) were also examined, as suggested by reference^45^. Exergy analysis focuses to calculate the amount of energy and explains the concept of irreversibility of the photovoltaic system with and without cooling (PCM/water).

Exergy balance analysis equation is similar to the energy balance analysis equation and Eqs. (13, 14) was used to compute the exergy balance of photovoltaic system with and without the use of PCM with water combination (cooling)^46^.

13 $${\dot{E}x}_{IN}={\dot{E}x}_{OUT}+{\dot{E}x}_{LOSS}$$

14 $${\dot{E}x}_{sun}+{\dot{E}x}_{mass, IN}={\dot{E}x}_{ele}+{\dot{E}x}_{mass, OUT}+{\dot{E}x}_{LOSS}$$

where, $\dot{E}$ x_LOSS_, $\dot{E}$ x_OUT_ and $\dot{E}$ x_IN_ represents the exergy loss, output and input rates respectively. The calculation of the rate of sun exergy involves the utilisation of incident solar irradiation to the system, which is expressed by Eq. (15).

15 $$\dot{E}{\text{x}}_{\text{sun}} = {\text{I}}\left(1-\frac{{T}_{amb}}{{T}_{sun}}\right)$$

where, I, ${T}_{amb}$ and ${T}_{sun}$ are the solar irradiance, ambient and sun temperature ${(T}_{sun}=5800K \; assumed \; as\; a \;black \;body)$. Equations (16) and (17) represents the thermal exergy $(\dot{E}$x_th_) of the photovoltaic system.

16 $$\dot{E}{\text{x}}_{\text{th}}={\dot{E}x}_{mass, OUT}-{\dot{E}x}_{mass, IN}$$

17 $${\left(\dot{m}{c}_{p}\right)}_{f}\left[{\left({T}_{out}-{T}_{in}\right)}_{f}-{T}_{amb}{\left(ln\frac{{T}_{OUT}}{{T}_{IN}}\right)}_{f}\right]$$

The exergy of the photovoltaic unit is equivalent to its electrical energy and can be determined through the utilisation of Eq. (18).

18 $${\dot{E}x}_{ele}={\dot{E}}_{ele}$$

The Exergy loss is given by Eq. (19) after substituting the Eq. (16–18) into Eqs. (13, 14).

19 $${\dot{E}x}_{LOSS}=I\left(1-\frac{{T}_{amb}}{{T}_{sun}}\right)-{\dot{E}}_{ele}-{\left(\dot{m}{c}_{p}\right)}_{f}\left[{\left({T}_{out}-{T}_{in}\right)}_{f}-{T}_{amb}{\left(ln\frac{{T}_{OUT}}{{T}_{IN}}\right)}_{f}\right]$$

Equation (20) is used to compute the rate of entropy generation, which is defined as the exergy loss to ambient temperature ratio.

20 $${\dot{S}}_{gn}=\frac{{\dot{E}x}_{LOSS}}{{T}_{amb}}$$

The efficiencies in electrical and thermal exergy of systems are determined by comparing the output electrical and thermal exergy to the total solar exergy over a specific timeframe. Hence, the expressions for electrical and thermal efficiencies can be formulated as follows^42^.

21 $${\varepsilon }_{el}=\frac{{\dot{E}x}_{el}}{{\dot{E}x}_{sun}}= \frac{{\dot{E}}_{el}}{(1-\frac{{T}_{amb}}{{T}_{sun}})}=\frac{{I}_{sc}{V}_{oc}FF}{{A}_{s}G}$$

22 $${\varepsilon }_{th}=\frac{{\dot{Ex}}_{th}}{{\dot{E}x}_{sun}}=\frac{{\left(\dot{m}{c}_{p}\right)}_{f}\left[{\left({T}_{out}-{T}_{in}\right)}_{f}-{T}_{amb}{\left(ln\frac{{T}_{OUT}}{{T}_{IN}}\right)}_{f}\right]}{G(1-\frac{{T}_{amb}}{{T}_{sun}})}$$

Moreover, the overall exergy efficiency of the system was computed as

23 $${\varepsilon }_{ov}= {\varepsilon }_{el}+{ \varepsilon }_{th}$$

### Evaluation of performance enhancement

The PV PCM system’s performance enhancement can be assessed using the specific parameters covered in this section. The parameters of power and efficiency percentage enhancement are denoted as the percentage difference in efficiency and power between a combined PV-PCM system and a conventional PV system. Hachem et al.^25^ suggested the Eq. (21) that provide the Efficiency enhancement and Eq. (24) the power enhancement percentage, respectively.

24 $$PEP=\frac{{P}_{O,PCM}-{P}_{O,ref}}{{P}_{O,ref}}\times 100\%$$

where, ${P}_{O,PCM}$, ${P}_{O,ref}$ are the output power from the PV-PCM/water system and conventional panel.

## Experimental programme

### Experimental setup

**Fig. 2:**
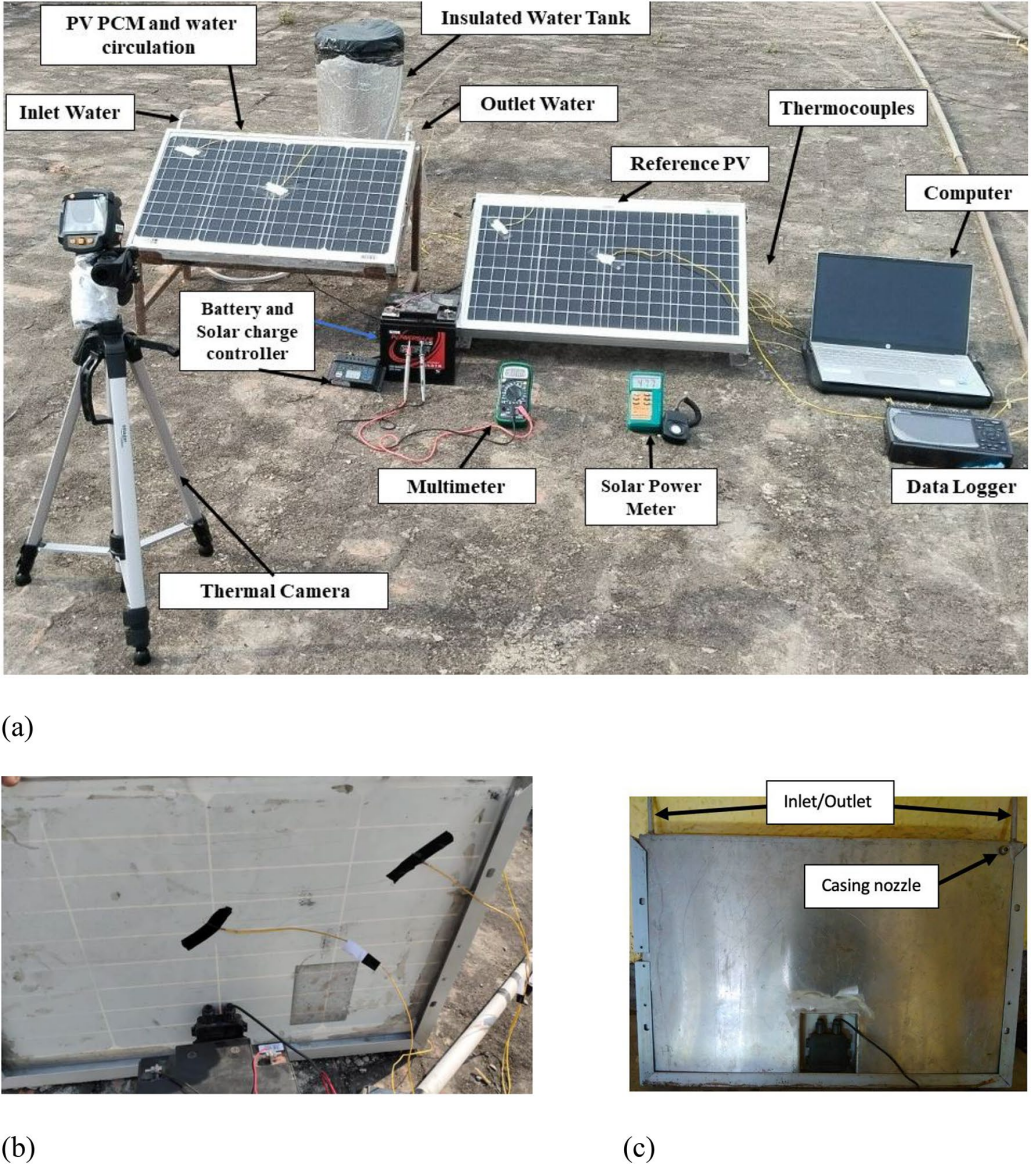
(**a**) Experimental test setup with cooling system, (**b**) back of PV without casing, (**c**) box casing fitted at the rare of PV indicating inlet and outlet of inbuilt pipe.

A 50 Wp monocrystalline PV panel is used for testing under real environmental conditions. The dimensions of the PCM casing are 660 × 460 × 20 mm, and the aluminium pipe is 20 mm in diameter and has three serpentine shaped loops. The complete set of PV panels, with or without a cooling agent, is installed on the roof to receive the sun’s radiation directly, and the surface temperature of each panel increases progressively after exposure to the sun. Figure 2 depicts the setup on the appropriate PV stand (made of MS material) at tilt angle 27° (facing south). The aluminium casing system containing the PCM are also shown in Fig. 2.

As indicated before, the experimental setup incorporates a phase change material (PCM) and a water absorber pipe within the PCM casing. To optimise the heat storage capacity of the phase change material (PCM) and enhance its heat evacuation and dissipation from the photovoltaic (PV) panel, a casing equipped with a water pipe flow system is affixed to the panel’s rear surface (i.e., the Tedler surface).

For data collection, the surface temperature of each PV panel (both the standard and the one with the cooling system) is continuously monitored using an array of calibrated thermocouples connected to a data logger system. This setup allows for precise measurement of temperature variations and helps in assessing the thermal performance of the PCM and water-cooling system.

Voltage and current measurements are taken using a calibrated multimeter, ensuring accurate electrical performance data for the PV panels. A solar power meter is used to measure the solar radiation intensity incident on the PV panels, allowing for correlation between solar irradiance and PV performance. The technical specification of the components used in this work are summarised in Appendix.

### Description

**Fig. 3:**
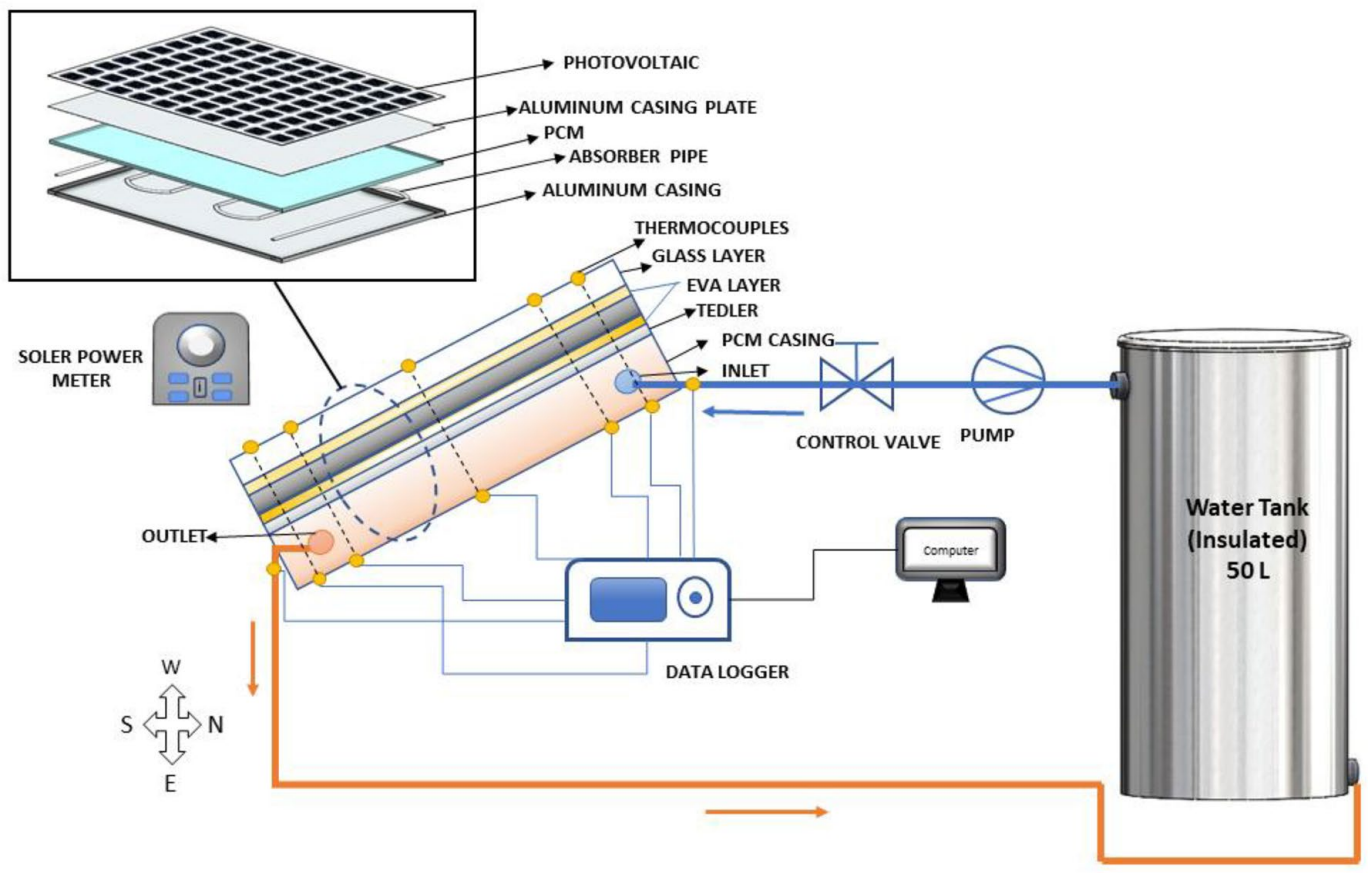
Schematic view of roof-top PV-PCM with water cooling system.

The experimental investigation of a PV panel incorporating a novel phase change material (PCM) and water heatsink combination is aimed at optimising the panel’s efficiency and mitigating the issue of high operating surface temperature. The PV panel is affixed with an aluminium casing that comprises a serpentine-shaped absorber pipe. This pipe is internally inserted in a manner that prevents contact with the container walls and is located at the back surface of the PV panel. A pipe’s key function is to enhance the PCM heat storage capacity by allowing water to flow through it. Figure 3 depicts the schematic view of the setup. The experiment was conducted at the Aligarh Muslim University (27.89°N latitude, 78.08°E longitude) in Aligarh (India) on the roof of the civil engineering department. Table 1 shows the specification of selected PV panel.

**Table 1:** Detail specification of photovoltaic panel.

Description	Specifications
Rated maximum power	50 W_p_
Electrical efficiency	16%
Short circuit current	3.08 A
Open circuit voltage	21.60 V
Material	Monocrystalline
Width	0.46 m
Length	0.66 m

The current study utilized the commercially available organic phase change material OM 30, which has a 30 °C phase transition temperature. OM 30 PCM temperature has a range (of ~ 23–30 °C), which is suitable for the current investigation’s geographical location of the study (Aligarh). A hot water bath positioned within a gas burner was used to indirectly raise the temperature of the PCM, which was obtained as powder or wax, to its phase transition temperature. The dissolved PCM is poured into the aluminium casing (made of industry grade) through a small opening on the rear of the casing, tightened with a screw and pushed into the frame at the back of PV panel (thereby easily detachable/replaceable architecture).

The (OM 30) PCM’s thermophysical properties are shown in Table 2. The phase change material (PCM) undergoes a transition from solid to liquid state as it absorbs heat (latent) energy from the aluminium casing (as depicted in Fig. 3), which is heated by the photovoltaic (PV) panel. The PCM then stores the heat energy as latent heat. The heat storage capacity of the Phase Change Material (PCM) for the purpose of eliminating surplus heat from the panel is sustained by the circulation of water through the cooling absorber pipe, which results in the dissipation of heat to the water circulation absorber pipe. The system benefits from improved thermal management and enhanced system performance.

**Table 2:** PCM (OM 30) thermo-physical properties.

Parameter	State	Value at 32–30 (°C)
Latent heat of fusion (kJ/kg)		230
Thermal conductivity (W/m K)	Solid	0.185
	Liquid	0.123
Density (kg/m^3^)	Solid	906
	Liquid	878
Specific heat capacity (J/kg K)		1260

### Instrumentation

During the experiment, the sun’s irradiance was tracked using a solar power meter (TM-207 TENMARS) with a range of 1999 W/m^2^ and an accuracy of 10 W/m^2^. The surface temperature of the cooled and uncooled photovoltaic systems was respectively measured with K-type thermocouple wires (K-type thermocouples have a range, accuracy, and sensitivity of -200 to 1250 °C, ± 2.2 °C, 41 µV/°C. The PV PCM system was equipped with four thermocouple wires, which were situated at both the top (upper) surface and the back (rear) of the system. The temperatures of the water entering and exiting the absorber pipe were recorded at the respective inlet and outlet points; the locations of thermocouple wires are shown in Fig. 3. All the calibrated thermocouple sensors are connected to a GRAPHTEC Data logger instrument (midi LOGGER GL840) to record each minute of temperature reading. A calibrated digital multimeter (MAS 830 series, MASTECH) was used to measure several electrical parameters, including open circuit voltage, short circuit current, maximum voltage, and maximum current, with a precision of ± 1.0%. The details and technical specification of these instruments were mentioned in the Table 8, Appendix.

### Procedure

During the experiment, a PCM-filled aluminium casing was used to dissipate the heat from the PV panel’s back surface. However, the PCM must maintain its latent heat storage capacity to remove more heat over a given period. The absorber pipe allows water to flow through to satisfy this requirement. In three distinct scenarios, the PV PCM system and water circulation through a water absorber pipe arrangement is the subject of experimental investigation. The performance of a solar photovoltaic system was analysed using measurements obtained from below mentioned three distinct experimental cases.

To start the trial (Case 1), an empty PCM aluminium casing, which is comparable in size to the PV board, is slid into the rear of the PV framework to eliminate heat from the board through normal convection and to assess the general presentation for 8 h over a period of 6 days.

The next stage (Case 2) utilized the same PV panel with the aluminium casing filled with PCM material. As the temperature of the top surface of the PV panel increases, the phase change material (PCM) undergoes a gradual melting with the absorption of heat. During peak hours, when the PCM is fully melted, it needs to maintain its heat storage capacity to store more latent heat, and this step is repeated for the same period of time.

In the final case (Case 3), a 12-V DC (8-Watt) water pump pushes water through the absorber pipe to maintain the PCM’s latent heat storage capacity and regulate the PCM’s temperature. To assess the electrical and thermal performance of the PV system during a given timeframe, two distinct mass flow rates, namely 0.0027 kg/s and 0.0034 kg/s, were employed on alternating days. A measuring cylinder and a timer were used to check that the water flow rate was constant before the experiment began. This was done to guarantee that the experiment’s various mass flow rates were carried out correctly. The experiment also tested the mass flow rate using the previously mentioned method. Throughout the experiment, the current study utilized a 50 L water tank.

## Uncertainty analysis

To ensure the reliability of the experimental results, uncertainty analysis on electrical, thermal efficiencies and power enhancement was analyse. Uncertainty analysis was conducted for the measured parameters and electrical efficiency. If *R* is a function of multiple independent variables ${X}_{1}$, ${X}_{2}$, …, ${X}_{n}$ the overall uncertainty ${U}_{R}$ of *R* can be calculated using the propagation of uncertainty formula^42^:

25 $${U}_{R}= \sqrt{{\left(\frac{\partial R}{\partial {X}_{1}}{U}_{{X}_{1}}\right)}^{2}+{\left(\frac{\partial R}{\partial {X}_{2}}{U}_{{X}_{2}}\right)}^{2}+ \cdots +{\left(\frac{\partial R}{\partial {X}_{n}}{U}_{{X}_{n}}\right)}^{2}}$$

Here, ${U}_{{X}_{1}}$ represents the uncertainty in the measurement of the variable ${X}_{i}$ , and $\partial R/(\partial {X}_{n} )$ is the partial derivative of R with respect to ${X}_{i}$.

The uncertainty analysis showed that the maximum power ${P}_{m}$ has an uncertainty of about ± 1.41%. We used the same method to calculate uncertainties for other parameters as well and presented in Table 3.

**Table 3:** Uncertainty from the experiment.

Parameters	Uncertainty
Electrical energy efficiency (flowrate 0.0027 kg/s)	± 0.1289%
Thermal energy efficiency (flowrate 0.0027 kg/s)	± 0.5376%
Overall energy efficiency (flowrate 0.0027 kg/s)	± 0.553%
Electrical energy efficiency (flowrate 0.0027 kg/s)	± 0.0529%
Thermal energy efficiency (flowrate 0.0027 kg/s)	± 0.0143%
Overall energy efficiency (flowrate 0.0027 kg/s)	± 0.055%
Power enhancement percentage	± 1.86%

## Results and discussion

### Experimental observations

In this section, experimental results for different cases of PV panels with and without cooling arrangements have been discussed.

**Fig. 4:**
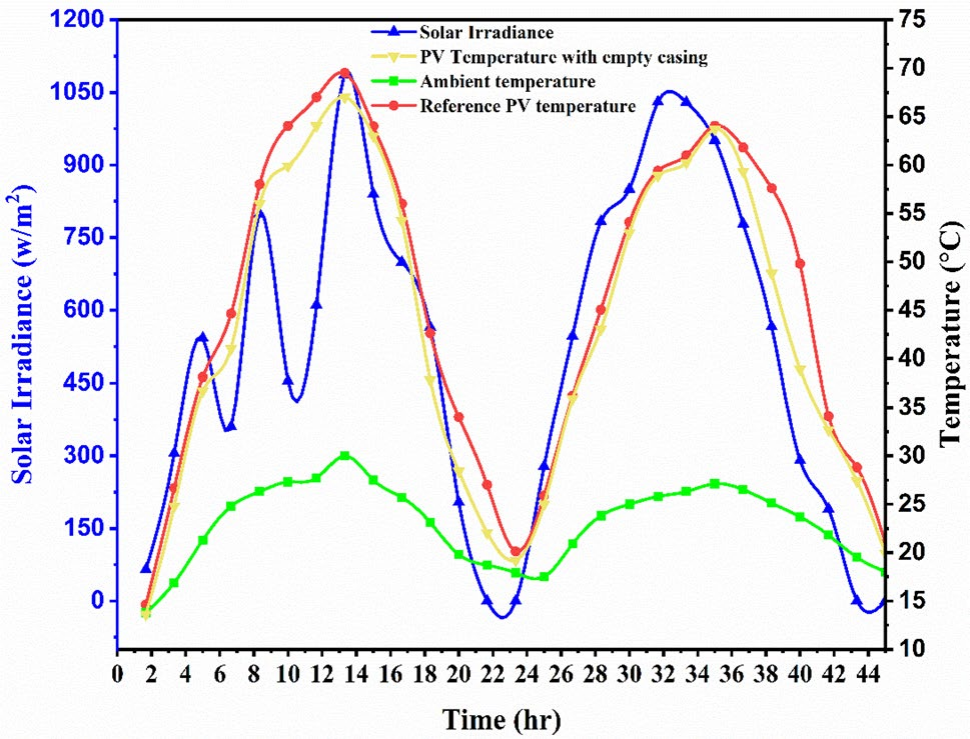
Variation in temperature behaviour of measured parameters for two days between the reference PV panel and the PV panel with empty casing.

The variations in the PV temperature readings which is attached to the empty casing, and reference panel at ambient temperature, and sun irradiance were measured during the two-day experiment between 6:00 and 19:00 h (plotted in Fig. 4). The graph indicates that Case 1’s average solar irradiance was 60 W/m^2^ at the beginning of the experiment and reached a peak of 1087 W/m^2^ between 10:00 and 13:00 h. The experiment ended with the lowest solar irradiance value, 0 W/m^2^. The graph shows that, the ambient temperature varies by 8.3 °C from the middle of the experiment to the end and by an average of 12 °C from the beginning to the middle of the experiment. Additionally, a conventional PV panel with an empty casing had a higher temperature at the upper (top) surface than the PV panel. The reference PV’s highest top surface temperature was 69.5 °C on the initial day and 64.05 °C on the second day at the same time (13 h), whereas the PV panel’s surface temperatures were 66.9 °C and 63.7 °C (Fig. 4). Throughout the experiment, the graph for Case 1 remained consistent overall.

**Fig. 5:**
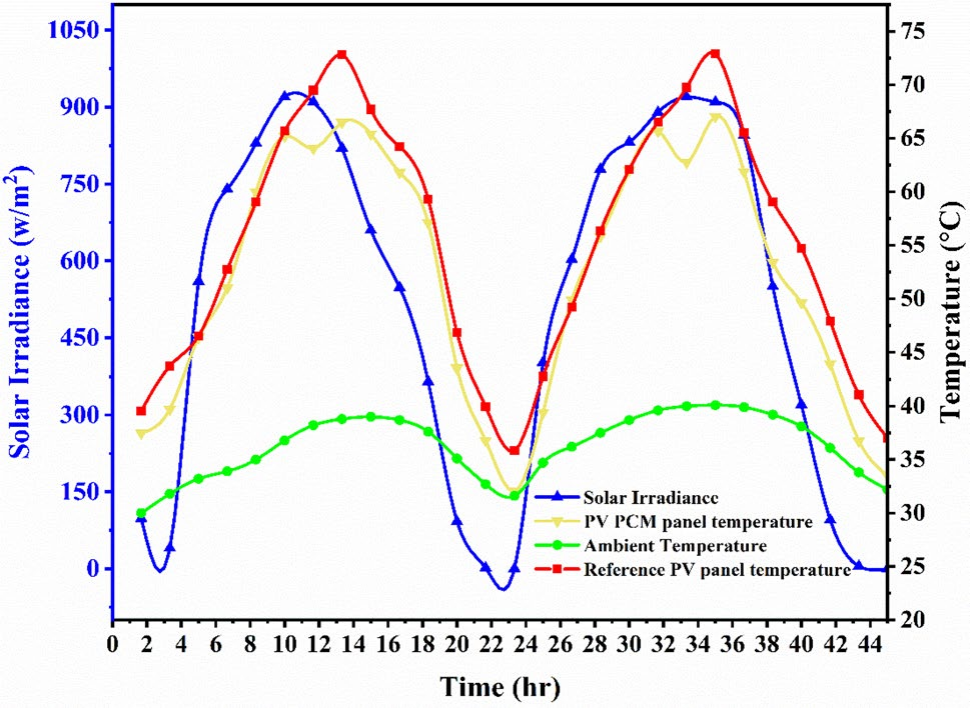
The temperature response of the measured parameters in the integrated PV PCM panel and the reference system varied by two days.

In the PV PCM system (Case 2), as depicted in Fig. 5, the introduction of PCM with the PV panel would have a significant effect compared to the conventional PV setup. From the beginning of the experiment, the average minimum temperature reduction was approximately 3.7 °C, and the maximum temperature reduction was approximately 5.6 °C between 12 and 15 h. From 8 to 11 h, phase change occurred, and it was observed that the top surface temperature increase of the integrated PV PCM setup was minimal (as shown in Fig. 5). As depicted in Fig. 5, the introduction of PCM with a PV panel would significantly lower the temperature as compared to the reference (uncooled) PV-board in the integrated PV PCM system (Case 2).

**Fig. 6:**
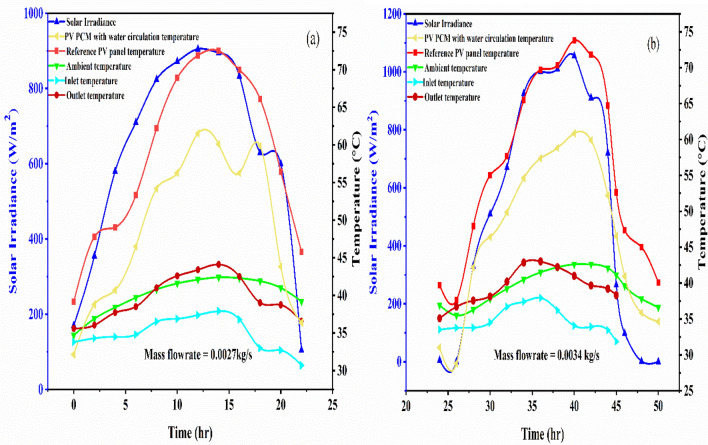
The temperature behaviour of the reference (conventional) PV panel and integrated PV PCM in combination with water at a mass flowrate of, (**a**) 0.0027 kg/s, and (**b**) 0.0034 kg/s.

Figure 5 also shows that the presence of PCM with water combination in the PV panel for Case 3a PV PCM panel aided with water circulation helped in reducing the temperature of the top surface of the PV system. From the beginning of the experiment, the average minimum result in temperature reduction was approximately 3.7 °C and the maximum result in temperature reduction was approximately 5.6 °C between 12 and 15 h. At the beginning of the experiment, a phase change occurred, and we observed the surface temperature of the PV panel. However, the top surface temperatures of the PV-PCM panel and the reference PV panel were comparable until there was no water flowing through the cooling channel in Case 2. From the beginning to the end of the experiment, a drop in the integrated PV-PCM top surface temperature was observed when the water flow was introduced compared to the reference PV panel top surface temperature. The highest top surface temperatures of reference PV and PV PCM with water circulation at a mass flow rate of 0.0027 kg/s are shown in graph of Fig. 6a. On the following day, in graph of Fig. 6b, the maximum top surface temperatures of reference PV and PV PCM with water circulation at a mass flowrate of 0.0034 kg/s were recorded at 73.8 °C and 60.05 °C during a peak time hour lasting 13 h. The variation of temperatures directly results from the intensity separated from the PV panel and utilised by a combination of PCM and water source combination. Additionally, it has been observed that temperature reduction slightly increased with a slight increase in mass flow rate. The photovoltaic panel’s surface temperature decreases further as the mass flow rate of the water increases. This is because the PCM maintains its latent heat capacity that absorbs more heat from the panel, and water flowing through pipe extracts the excess heat from PCM. The measurements shown in graph 6a and 6b were carried out on consecutive days, and the separate subplots were used for clarity in distinguishing flowrate effects.

### Energy analysis

To thoroughly analyse the various scenarios investigated in this experiment concerning system performance, Table 4 summarizes the averages of electrical and thermal power, along with electrical and thermal energy efficiency, across: conventional PV modules, PV/PCM configurations, and PV/PCM systems integrated with water circulation at two distinct flow rates. Observing the table, it is evident that employing the PV/PCM with water circulation system enhances both electrical power and electrical energy efficiency. Specifically, the average increase in electrical energy efficiency for the PV/PCM with water circulation at a flow rate of 0.0034 kg/s compared to the standalone PV unit is 3.65%. This underscores the substantial impact of integrating a fluid circulation pipe with PCM in the system, as the fluid flow aids in better heat absorption compared to using PCM alone. The integration allows for efficient heat extraction from the PCM, maintaining its latent heat storage capacity, consequently enabling greater heat absorption from the panel and sustained temperature reduction over extended periods, thereby enhancing output power. Notably, Table 4 illustrates that the PV/PCM with water circulation system demonstrates superior electrical energy and electrical energy efficiency compared to other configurations.

**Table 4:** The average electrical, thermal energy and efficiencies of the system.

System type	PV/empty casing system		PV/PCM system		PV/PCM with water circulation system			
Parameters (average values)	Conventional PV	Empty Casing	PV/ PCM	Conventional PV	Flowrate 0.0027 kg/s	Conventional PV	Flowrate 0.0034 kg/s	Conventional PV
Electrical energy (W/m^2^)	19.02	20.7	22.8	18.2	22.76	18.3	24.26	17.8
Electrical energy efficiency (%)	9.94	11.02	12.8	10.4	12.89	10.07	13.75	10.75
Thermal energy (W/m^2^)	–	–	–	–	53.18	–	53.5	–
Thermal energy efficiency (%)	–	–	–	–	53.76	–	74.04	–
Overall energy efficiency (%)	9.94	11.02	12.8	10.4	66.65	10.07	87.79	10.75

Information detailed in Table 5 presents the maximum overall thermal and electrical exergy outputs for both the reference PV panel and the PV-PCM system with water circulation across all cases. Across all experiments, the average electrical exergy for the reference PV panel was approximately 73.5 W/m^2^. Notably, the electrical exergy of the PV panel utilizing PCM and water circulation demonstrated a higher value compared to the reference PV panel. Table 5 illustrates a 1.57% higher electrical exergy efficiency over a two-day span for the PV/PCM with water circulation system in contrast to the standalone PV unit. Integrating a collector with PCM significantly impacted system performance by facilitating enhanced heat absorption from the PCM through fluid flow, thereby enabling better heat absorption from the system compared to using PCM alone.

**Table 5:** The average electrical, thermal exergy and efficiencies of the system.

System type	PV/empty casing system		PV/PCM system		PV/PCM with water circulation system			
Parameters (average values)	Two days Conventional PV	Two days Empty Casing	Two days PV/PCM	Two days Conventional PV	Flowrate 0.0027 kg/s	Conventional PV	Flowrate 0.0034 kg/s	Conventional PV
Electrical exergy (W/m^2^)	19.02	20.7	22.8	18.2	22.76	18.3	24.26	17.8
Electrical exergy efficiency (%)	6.01	6.68	7.91	6.29	5.29	3.08	4.19	3.18
Thermal exergy (W/m^2^)	–	–	–	–	45.49	-	45.55	–
Thermal exergy efficiency (%)	–	–	–	–	1.43	-	1.35	–
Overall exergy efficiency (%)	6.01	6.68	7.91	6.29	6.72	3.08	5.54	3.18

Additionally, the maximum thermal exergy differed between flow rates of 0.0034 kg/s and 0.0027 kg/s. The average thermal exergy efficiency for these flow rates stood at 6.72% and 5.45%, respectively. These outcomes underscore the influential role of thermal regulations in affecting the exergy efficiency of electrical energy. The overall exergy efficiencies across Case 1 to Case 3 were 6.68%, 7.91%, 6.72%, and 5.45%, respectively. The integration of PCM with water circulation within the PV panel demonstrated commendable thermal stability and ensured efficient utilization of extracted thermal energy for various applications. The comparison of this experimental value of overall exergy efficiency with the works of Sudhakar et al.^42^, Kazemian et al.^41^ and Xu et al.^36^ reveals noticeable differences. Our case involves the fluid pipe solely absorbing PCM heat, while in the literature, the pipe is attached to the casing, enabling it to absorb heat from the entire system. Consequently, our thermal exergy efficiency is comparatively lower than the literature’s values (refer Table 5).

### Performance of power output

**Fig. 7:**
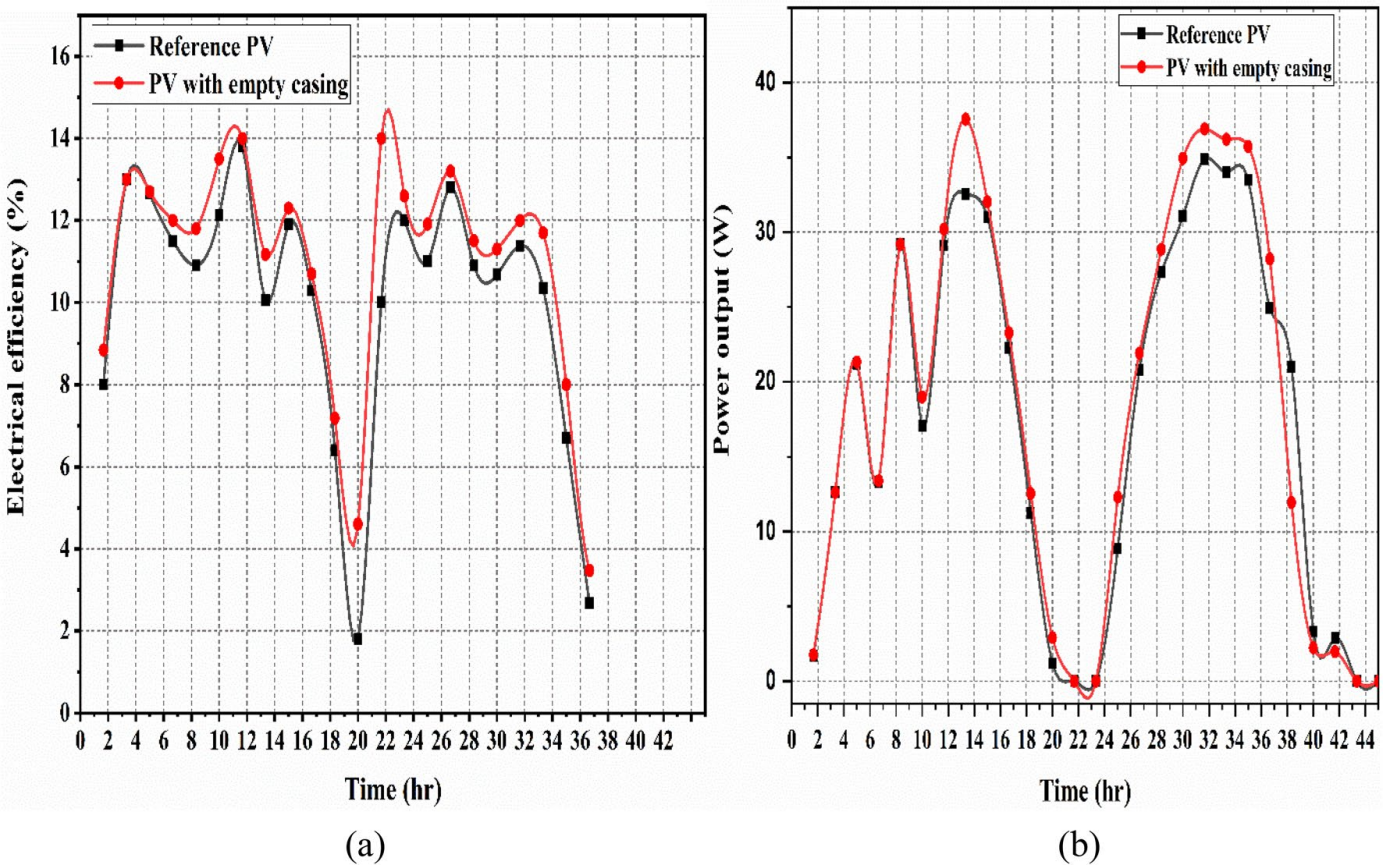
The empty-case PV and Reference PV’s, (**a**) electrical efficiency, and (**b**) the output power.

The generation of electrical power and efficiency of each case are compared in this part. Figure 7a and b depict the analyses of electrical efficiency (referred to Eq. 11) and power output (referred to Eq. 3) of a PV-panel with an empty casing and a reference PV panel for Case I. The reference PV panel and the PV panel with empty casing attained an electrical efficiency average of 11.02% and 9.94%; that respectively produced an average electricity of 19.02 W and 20.7 W (mentioned in Table 4). During the two-day testing period (see Fig. 7), the reference PV panel’s electricity generation and electrical efficiency were nearly identical to those of the PV fitted with an empty casing at the initial of the experiment due to the absence of PCM and water flow.

**Fig. 8:**
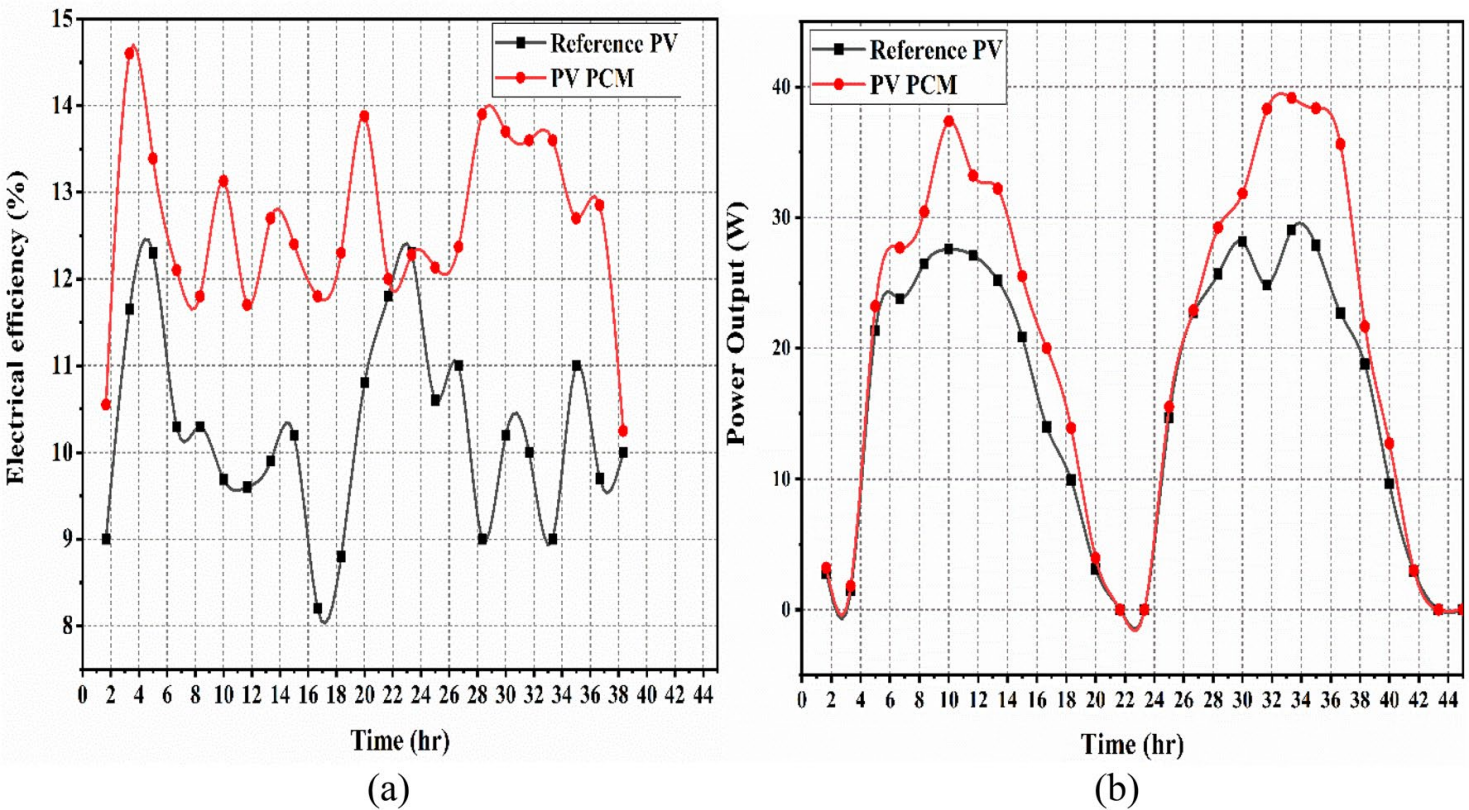
Reference PV’s and PV PCM’s, (**a**) electrical efficiency, and (**b**) power output.

For Case II, Fig. 8a and b and Table 4 present a comparison of the electrical efficiency and power output between the integrated PV PCM system and the PV panel without cooling attachment. The integrated PV PCM panel system and the without cooling attachment PV panel had an average maximum efficiency of 12.8% and 10.4%, respectively. In contrast, the integrated PV PCM and the conventional PV systems had an average maximum power output of 22.08 W and 18.2 W, respectively. This is mainly because the PCM filled aluminium casing absorb heat from the panel, thereby, yielding better efficiency than Case I. In addition, the initial power output graph was comparable due to the absence of flow of the water in the cooling arrangement channel.

**Fig. 9:**
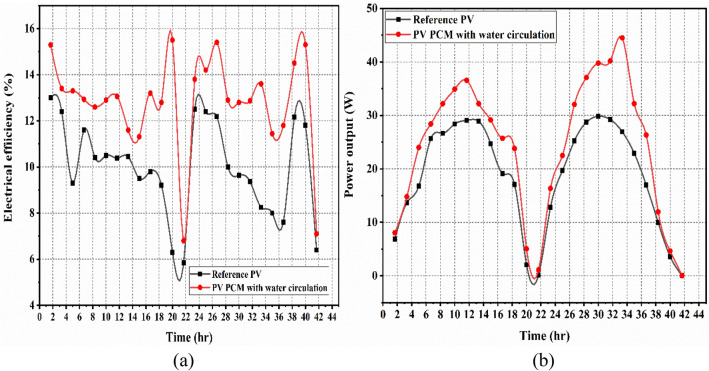
Reference PV’s and PV PCM’s with water circulation, (**a**) electrical efficiency, and (**b**) power output.

Figure 9a and b and Table 4 present a comparison of the electrical efficiency and power output between a PV panel with PCM and water circulation (PV PCM with water circulation) and a reference (uncooled) PV panel for Case III. The utilisation of a phase change material (PCM) chamber aided with water circulation (mass flowrate 0.0027 kg/s and 0.0034 kg/s) in photovoltaic (PV) panel resulted in an average maximum efficiency of 12.89%. In comparison, the reference PV panel achieved an average maximum efficiency of 10.07% at water flowrate of 0.0027 kg/s. While, the PV PCM water circulation combination and the reference PV system had an average maximum power output of 22.67 W and 18.3 W, respectively. A mass flowrate of 0.0034 kg/s resulted in an average maximum efficiency of 13.75%. In comparison, the reference PV panel achieved an average maximum efficiency of 10.75%. And the PV PCM water circulation combination and the reference PV system had an average maximum power output of 24.26 W and 17.8 W, respectively. It has been observed that the electrical efficiency and power output of the system exhibit an increase as the water mass flow rate is increased. An expansion in the mass stream pace of water builds the cooling of the photovoltaic board, which straightforwardly influences its electrical productivity and power. This was because the combination of the PCM and water circulation retained the PCM’s latent heat storage capacity while extracting more heat from the PV panel’s surface, particularly from the water flow. As a result, the PV system’s performance improved in this instance.

Table 6 illustrates the impact of the power necessary for fluid pumping through the serpentine-type pipe on the electrical power and electrical energy efficiency within the PV/PCM water circulation system. As indicated before, in this experimental setup, a pump is employed to circulate the working fluid within the PCM casing, utilizing two distinct mass flow rates: 0.0027 kg/s on the first day and 0.0034 kg/s on the subsequent day. The calculation of the energy needed for pumping the fluid through the systems is performed using Eq. $(10)$. As shown in the table, the influence of pumping power on both output electrical power and electrical energy efficiency for both flow rates are below 1%. Consequently, the energy required for fluid pumping within the PV/PCM water circulation system has been disregarded in the study.

**Table 6:** Investigation of the effect of required power for pumping on electrical power and electrical energy efficiency of the PVT/PCM system.

PV/PCM-water system (flowrate):	0.0027 kg/s	0.0034 kg/s
${\dot{E}}_{pump}$	0.0075	0.0011
${\dot{E}}_{el}$	22.761	24.269
${\dot{E}}_{net,pump}$	22.748	24.260
${\eta }_{el}$	12.89	13.75
${\eta }_{net,el}$	12.84	13.53

A comparison of our study was done with the study provided by Sudhakar et al.^22^ the use of PV PCM with water combination gives an average electrical efficiency enhancement of 12.4% and an average surface temperature reduction of 5.4 °C. A similar study was done by Preet et al.^21^, and their results showed a 12.6% increment in electrical efficiency using a mass flow rate of 0.031 kg/s. While in our improved design, using PV PCM and water circulation, maximum electrical efficiency enhancement was 13.75% and the temperature reduction was 5.6 °C. Hence it is mainly the positioning of coolant pipes that contribute to the direct heat transfer from the PCM and enhances the storage capacity of latent heat by the given amount of PCM; thereby, also saving on the quantity of PCM required in comparison to the case where coolant is not supplied through the embedded pipes. Table 7 presents the findings from this research alongside those from prior studies focusing on various PV/PCM water circulation systems.

**Table 7:** Summary of the comparison improvement of the surface temperature and Electrical efficiency of the PV/PCM water circulation system.

System type	Surface temperature reduction (°C) and (%)	Electrical efficiency achieved (%)	Remark	References
PVT-PCM	53%	12.6	Pipe was directly attached with casing (flowrate 0.031 kg/s)	Preet et al.^35^
PVT-PCM	20.42 °C	14.027	Pipe was directly attached with casing (constant flowrate 30 kg/h)	Kazemien et al.^41^
PV-PCM water casing	5.4 °C	12.4	Water cooling chamber was attached behind the PCM (constant flowrate 0.00077 kg/s)	Sudhakar et al.^42^
PVT/PCM	50–45 °C	12.6–13.7	Water flow pipe was attached with casing (flowrate 1.7 L/min)	Xu et al.^36^
PV/PCM-water circulation	5.6 °C	13.75	Water flow pipe was within the casing without touching internally (flowrate 0.0034 kg/s)	Present Study

### Power enhancement

**Fig. 10:**
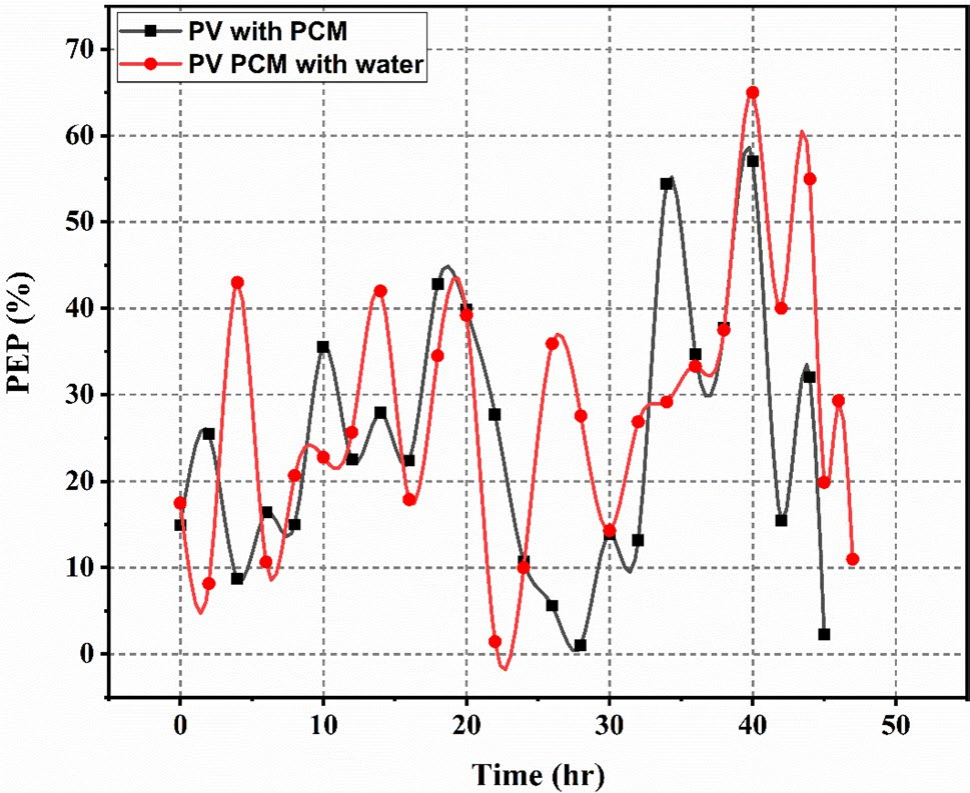
Percentage of power enhancement of PV supported by PCM only and PV supported by PCM with water circulation.

The percentage difference between the power generated by PV PCM and PV PCM with water circulation and the conventional PV system during the period of experiments was used to calculate the power enhancement percentage. Figure 10 depicts the PV-PCM panel’s power gain in each scenario. The experiments’ power enhancement percentages were positive because the PV-PCM with water circulation generated more power than the Reference PV Panel. Up until 11:30 h, the experiment’s power was not significantly increased. However, because the PCM’s latent heat capacity is maintained by the water flowing through the cooling pipe, a notable rise in the percentage of power improvement was observed. The experiment’s power enhancement performance increased between 13:30 and 14:00 h, as depicted in the figure. The PV-PCM water circulation case’s average power enhancement was 24.04%, while the PV-PCM panel’s average was 27.6%.

### Loss of exergy and entropy generation

**Fig. 11:**
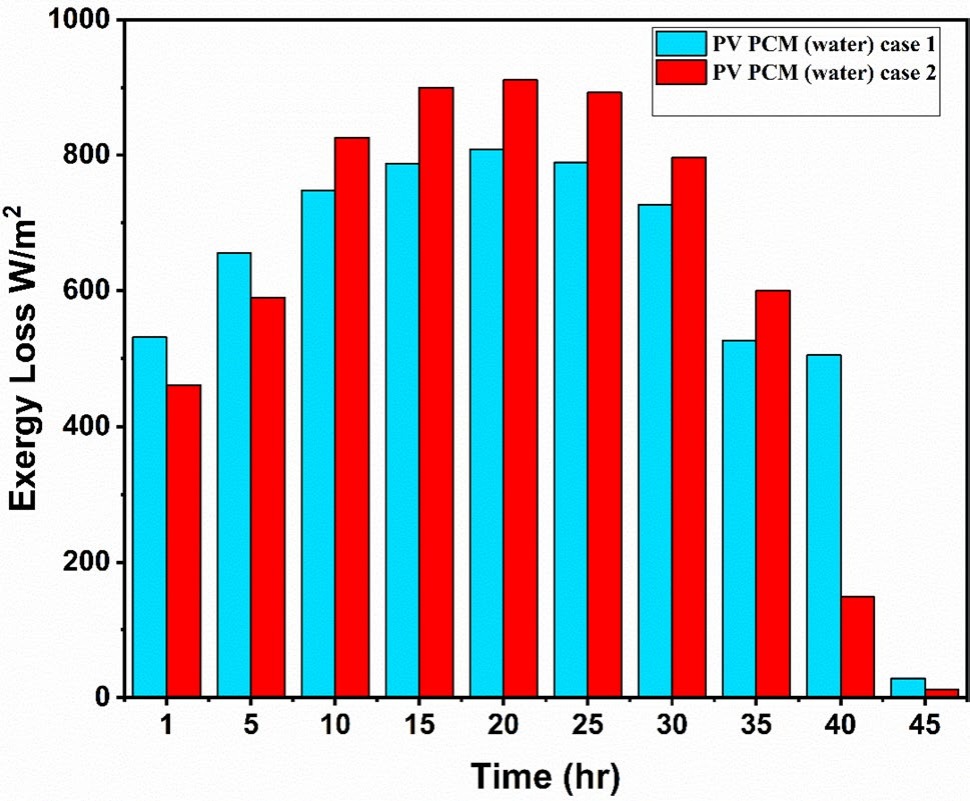
Exergy loss during the experimentation.

**Fig. 12:**
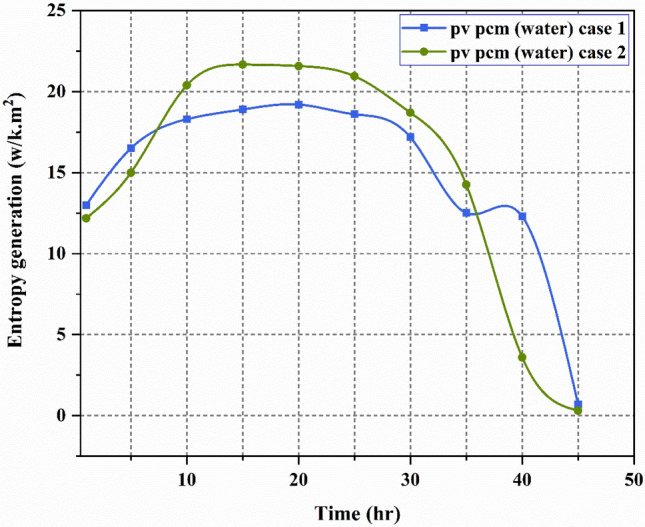
Entropy generation during the experimentation.

Figures 11 and 12, respectively, depict the PV-PCM panel’s exergy loss and entropy variation for the experiment (Case I with PCM and water Circulation). The data in figures indicates that the PV-PCM panel utilised in the experiments exhibits entropy generation that is consistent with exergy loss. The PV-PCM panel’s loss of exergy and entropy generation for Cases I and II increased continuously until noon, reaching maximum values of 808.9 W/m^2^, 19.2 W/K m^2^ and 911 W/m^2^, 21.58 W/K m^2^ respectively, before decreasing till the conclusion of the experimental work. As the experiment progressed toward noon, the sun’s exergy increased which is the cause of the variation.

The study indicates that the input exergy experiences a decline in the afternoon, resulting in the attainment of its minimum values at the conclusion of the experiment, namely 28.49 W/m^2^, and 0.7 W/K m^2^ for loss of exergy, respectively. The corresponding values for loss of entropy generation are 12.14 W/m^2^, and 0.306 W/K m^2^. Similar tendency was seen in the variances in exergy loss and entropy generation by Sudhakar et al.^42^

The experimental results indicate that an improvement in heat transfer from the phase change material (PCM) to the water circulation led to a reduction in the top surface temperatures of the photovoltaic-PCM (PV PCM) panels. The findings suggest that 0.0034 kg/s of the mass flowrate during the experiments resulted in a more effective thermal management of the PV-PCM panel with water circulation in the cooling pipe, as compared to the conventional photovoltaic system. Although the findings of the study also present a key enhancement in both thermal management and electrical efficiency of PV panels through the incorporation of PCM and water circulation systems. These results hold practical significance for rooftop PV installations, particularly in regions characterized by semi-arid and hot climates. Specifically, our integration of PCM with a melting point of 30 °C and water circulation demonstrates effective reduction in PV panel operating temperatures, thereby offering potential for improved efficiency and prolonged panel lifespan in high ambient temperature environments. Moreover, the observed increase in electrical efficiency, reaching up to 13.75%, suggests that PV systems utilizing our PCM-water cooling design have the capacity to generate greater electricity output from the same installed capacity, thus enhancing overall economic viability. The design of our system, which integrates a serpentine pipe within the PCM casing without direct contact with internal walls, presents a practical and efficient solution for heat exchange, facilitating ease of installation and handling in both new and retrofit applications.

The deviation between current results and the existing literature are compared and justified.

### Surface temperature reduction

Observed deviation: In present study, the surface temperature reduction was 5.6 °C, which is comparable to the 5.4 °C reduction observed by Sudhakar et al.^42^. However, it is significantly lower than the reductions reported by Preet et al.^35^ and Xu et al.^36^, which ranged from 50 °C to 53%.

The higher temperature reductions reported in other studies can be attributed to the direct attachment of the cooling pipe to the casing, which enhances heat transfer efficiency. In our study, the serpentine pipe is integrated within the PCM casing without direct contact with the internal walls, optimizing heat transfer while avoiding localized heating. This design choice improves the system’s overall performance but results in a lower temperature reduction compared to direct attachment methods.

### Degradation and instability of PCM

The proposed PCM water circulation cooling system demonstrated significant short-term enhancement in PV performance, the long-term durability of PCM is an ongoing issue. The literature mentions that there are several potential issues with PCM, such as phase separation, subcooling, leakage, and material degradation that have been reported during successive melt-solidification cycles that may introduce thermal reliability issues with PCM. These phenomena are likely to ultimately impact the cooling efficiency and effectiveness and system efficiency at longer operating conditions. Due to the controlled phase and relatively short cycles in the experimental evaluation, no apparent degradation in PCM properties was observed. Future studies will be required to evaluate phase change material integrated with serpentine-pipe casing for cyclic stability, in order to fully explore the practicality of applications on large-scale roofs under semi-arid conditions.

### Electrical efficiency improvement

Observed deviation: The present study achieved an electrical efficiency improvement of 13.75%, which is higher than the 12.4% reported by P. Sudhakar et al.^42^ and within the range reported by Xu et al.^36^ (12.6–13.7%).

The higher electrical efficiency in our study can be explained by the optimal placement and flow rate of the serpentine pipe within the PCM casing. The flow rate of 0.0034 kg/s was found to be effective in maximizing heat transfer from the PCM, thereby maintaining a lower PV panel temperature and enhancing electrical efficiency. This approach contrasts with the lower flow rate used by Sudhakar et al.^42^, which may have resulted in less efficient cooling.

### Power output enhancement

Observed deviation: The power output enhancement in the present study was 24.14 W, which is not directly comparable to the other studies as they did not explicitly report this parameter.

The power output enhancement is a direct result of the improved electrical efficiency achieved through our optimized cooling system design. The integration of the serpentine pipe within the PCM casing without touching the internal walls allows for more uniform heat distribution and efficient heat removal, leading to increased power output.

In this study, wind speed was not measured directly. The testing was performed under outdoor natural conditions, where the effect of wind was not disrupted. It is well known that air velocity has a significant effect on convection heat transfer from the PV surface. However, since the same ambient conditions were applied to all models tested, the reported relative performance trends remain valid. Wind speed measurements will be incorporated in future work to improve the analysis.

## Conclusion

The study examines the efficacy of roof-mounted PV cooling with PCM material (OM 30) and water combinations to lesser the high operating PV surface temperature and enhance the system’s electrical performance. The obtained consequences (result) were compared to those of the reference PV system that did not have any cooling arrangements. The outcomes demonstrated that the PCM-equipped PV panel and water circulation (Case III) (flowrate 0.0034 kg/s) improved the heat dissipation rate of the PV, which further improved the system’s power enhancement as opposed (compared) to the system with PCM and an empty casing (Case II). With only PCM cooling (Case II), the PV’s maximum surface temperature was approximately 67.05 °C; however, when PCM was assisted with the circulation of water, the PV surface temperature was 60.08 °C at a mass flowrate of 0.0027 kg/s, and a slight increase in mass flow rate (0.0034 kg/s) resulted in a temperature of approximately 60.05 °C. Increasing the mass flow rate increases heat dissipation (with pump energy consumption was negligible) and preserves the PCM’s capacity for latent heat storage, enabling it to draw out more heat from the photovoltaic panel’s surface. Furthermore, the performance metrics are assessed and reported, including power generation, analysis of exergy, power efficiency for the photovoltaic panel, and power increase performance factor. The key observations include the following:

- The maximum increase in electrical efficiency for the PV/PCM system with water circulation was determined to be 13.75%, accompanied by a temperature reduction of 5.6 °C. This case was identified as the best in terms of overall system performance compared to other cases because the energy consumed by the pump was negligible.
- The average power enhancement of the PV-PCM water circulation case was found to be 24.04%, while the average power enhancement of the PV-PCM panel was 27.6%.
- The average electrical exergy and exergy efficiency of the PV PCM with water circulation at flowrate 0.0034 kg/s were improved by 6.46% to 1.01%, respectively.
- The exergy loss and entropy generation of the PV-PCM panel exhibited a continuous increase for Cases I, II and III, ultimately peaking at 808.9 W/m^2^, 911 W/m^2^, 870.8 W/m^2^, and 19.2 W/K m^2^, 21.58 W/K m^2^, 22.7 W/K m^2^ respectively, until noon. According to the findings of the study, there is a decrease in the input exergy during the afternoon.

The current system configuration exhibits an advancement in comparison to prior literature, primarily attributed to the incorporation of water circulation pipes that are embedded within the PCM casing, without making contact with the casing walls. This facilitates the direct transfer of heat from the PCM, thereby augmenting the latent heat storage capacity by a specific amount of PCM.

Although these achievements have been recognized, some limitations should still be noted. The long-term stability and degradation of PCMs under a series of charge–discharge cycles have not been investigated. Wind velocity, which may affect convective cooling, was not measured during the experiments and should be part of future studies. Furthermore, the experimental tests were performed under specific climatic conditions (semi-arid, Aligarh, India) and results may vary in other environmental scenarios. Future research direction works include optimizing PCM materials and water circulation systems for further efficiency gains, assessing long-term durability, and exploring alternative cooling strategies. Addressing these areas will contribute to advancing PV thermal management and enhancing solar energy technologies. Conduct a comprehensive techno-economic analysis will be essential to assess the feasibility and cost-effectiveness and payback period of the proposed cooling system for different applications and locations and develop the optimization algorithms to determine the optimal design parameters, such as PCM type, water flow rate, and pipe configuration, based on specific site conditions and performance objectives will provide practical guidelines for scaling up this technology. Addressing these aspects will contribute to advanced Photovoltaic thermal management system and also improving the economic viability of the system.

## Data Availability

Data is available on reasonable request made to author Mohammad Uzair.

## Appendix

See Table 8.

**Table 8:** Technical specification of the components and instruments.

Technical specification of the components used in this study		Standard uncertainties, (k = 2)
Solar photovoltaic panel		
Rated maximum power	50 Wp	–
Electrical efficiency	16%	**–**
Short circuit current	3.08 A	–
Open circuit voltage	21.60 V	**–**
Current at Pmax	2.81 A	–
Voltage at Pmax	17.80 V	**–**
Nominal operating cell temperature	45 ± 2 ֯C	–
Maximum series fuse rating	06 A	**–**
Material	Monocrystalline	–
Width	0.46 m	**–**
Length	0.66 m	–
Solar power meter		
Range	2000W/m^2^, 634BTU/(ft^2^ × h)	**–**
Resolution	0.1W/m^2^, 0.1 BTU/(ft^2^ × h)	–
Accuracy	± 10W/m^2^ [± 3BTU/(ft^2^ × h)] or ± 5% whichever is greater in sunlight. Temperature included error ± 0.38W/m^2^/°C [± 0.12BTU/(ft^2^ × h)]/°C] deviation from 25 °C	± 5%
Drift	less than ± 2% per year	**–**
Sampling rate	0.25/second	–
Operating temperature	0 °C to 50 °C, humidity below 80% RH	**–**
Graphtec data logger GL840		
Number of analog input channels	20 channels in standard configuration	± 0.02 mV
Number of analog input terminals	Up to 10 terminals (20 channels/terminal)	**–**
Input voltage range	20 mV to 100 V	–
Thermocouple	R, S, B, K, E, T, J, N, W (WRe5-26)	**–**
RTD (resistance temp. detector)	Pt100 (IEC751), Pt1000 (IEC751), JPt100 (JIS)	–
Accuracy	± 0.1% of F.S. (full scale)	**–**
Battery		
Model	EP26-12	–
Type of product	Solar battery (EXIDE)	**–**
Voltage	12 V	–
Battery capacity	26 Ah	**–**
Battery cell composition	Lead-acid	–
Solar charge controller		
Rated voltage	12 V/24 V	**–**
Rated current	40A	**–**
Max PV voltage	50 V	**–**
Max. PV input power	520W(12 V)/1040W(24 V)	**–**
DC pump		
Voltage	12 V	**–**
Power	8w	**–**
Head-max	5 m	**–**
Flow rate	10 L/min	**–**
Thermocouple		
Type	K	± 0.1 °C
Accuracy	± 2.2 °C	**–**
Temperature range	− 200 to 1250 °C	**–**
Sensitivity	41 µV/°C	**–**
Digital multimeter	± 0.5% V, ± 1% A	± 0.01 V, ± 0.01 A

## List of symbols

${A}_{s}$: PV surface area (m^2^)
*C*_*p*_: Specific heat capacity (J kg^−^^1^ K^−^^1^)
$$\dot{E}$$_in_: Input energy (W)
$$\dot{E}$$_out_: Output energy (W)
$${\dot{E}}_{loss}$$: Loss energy (W)
$\dot{E}$_s, pv_: PV cell stored energy (W)
$$\dot{E}x$$: Exergy rate (W)
${\dot{E}}_{el}$: Electrical power output from the system (W)
${\dot{E}}_{pump}$: Power need for pump (W)
$${\dot{E}}_{th}$$: Thermal energy (W)
*G*: Solar irradiance (W/m^2^)
${\dot{G}}_{beam}$: Beam solar irradiance (W/m^2^)
${\dot{G}}_{diffuse}$: Diffuse solar irradiance (W/m^2^)
*I*: Electrical current (A)
$${I}_{s}$$: Sun hours (W/m^2^)
$\dot{m}$: Mass flow rate (kg/s)
*P*: Power output (W)
${P}_{O,PCM}$: Power output PCM (W)
${P}_{O,ref}$: Power output reference panel (W)
$\dot{Q}$: Convective heat transfer (W/m^2^)
$$\dot{S}$$: Entropy generation (W/k.m^2^)
*T*: Temperature (°C)
*V*: Voltage (V)

## Greek letters

*α*: Absorptivity of solar PV panel
$\eta$: Efficiency of the PV panel/pump (%)
$\tau$: Transmittivity of glass layer of the PV
$$\rho$$: Density (kg m^−^^3^)
$$\varepsilon$$: Exergy efficiency (%)
$$\Delta P$$: Pressure drop

## Subscript

amb: Ambient
el: Electrical
$$\text{f}$$: Fluid
gn: Generation
in: Inlet
out: Outlet
O: Output
ov: Overall
oc: Open circuit
PCM: Phase change material
ref: Reference
s: Surface
sc: Short circuit
th: Thermal

## Abbreviations

BIPV: Building integrated photovoltaic
DC: Direct current
EG: Ethylene glycol
FF: Fill factor
GNP: Graphene nano particle
MEPCM: Micro encapsulated phase change material
MS: Mild steel
NEPCM: Nano encapsulated phase change material
PV: Photovoltaic
PCM: Phase change material
PVT: Photovoltaic thermal
RT: RubiTherm
UAE: United Arab Emirates
Wp: Watt power
